# Analysis of Adherence to Acute Inpatient Rehabilitation in Patients with Cancer

**DOI:** 10.7150/jca.61010

**Published:** 2021-08-20

**Authors:** Jegy M. Tennison, Carly M. Sullivan, Brian C. Fricke, Eduardo Bruera

**Affiliations:** 1 Department of Palliative, Rehabilitation, and Integrative Medicine, The University of Texas MD Anderson Cancer Center, Houston, Texas.; 2 Department of Rehabilitation Services, The University of Texas MD Anderson Cancer Center, Houston, Texas.; 3 Department of Rehabilitation Medicine, UT Health San Antonio Long School of Medicine, San Antonio, Texas.

**Keywords:** neoplasm, inpatients, guideline adherence, exercise, occupational therapy, rehabilitation

## Abstract

**Importance:** The need for cancer rehabilitation is expected to continue to dramatically increase with the aging population and increasing number of cancer survivors. These survivors experience a wide range of physical limitations and symptoms that negatively affect their health and quality of life. Research is needed to determine the rate of adherence, reasons for non-adherence, and interventions to improve adherence to acute inpatient rehabilitation among patients with cancer.

**Objective:** To evaluate the rate of adherence and reasons for non-adherence to acute inpatient rehabilitation in patients with cancer.

**Design, Setting, and Participants:** This was a secondary analysis of a retrospective study that assessed medical complications in 165 patients with cancer who had a median length of stay of 11 days (interquartile range of 8-14) in acute inpatient rehabilitation. We reviewed the records of all consecutive patients who underwent acute inpatient rehabilitation from September 1, 2017 through February 28, 2018 at a large academic, quaternary National Cancer Institute-designated Cancer Center.

**Main Outcomes and Measures:** We calculated the rehabilitation session adherence rate and descriptively summarized the reasons for non-adherence.

**Results:** There were 78/165 (47%) patients that had 1 or more incomplete rehabilitation sessions due to medical complications. These patients had a median of 2 (interquartile range of 1-4) incomplete rehabilitation sessions. We noted other reasons for incomplete rehabilitation sessions in 146/165 (89%) patients, who had a median of 3 (interquartile range of 2-4) incomplete rehabilitation sessions. The median total number of days with incomplete rehabilitation sessions in the entire cohort was 2 (interquartile range 1-3).

**Conclusion and Relevance:** Among patients with cancer undergoing acute inpatient rehabilitation, the adherence rate to 1-hour long intensive rehabilitation sessions were low due to medical complications and other reasons. This in turn affected compliance with the 3 hours of rehabilitation a day requirement for acute inpatient rehabilitation. Patients with cancer undergoing acute inpatient rehabilitation are medically complex and further research at multiple institutions with larger cohorts may be beneficial in further assessing adherence rates and reasons for non-adherence to improve participation in acute inpatient rehabilitation.

## Introduction

For admission to acute inpatient rehabilitation, the Centers for Medicare & Medicaid Services (CMS) require that the patient 1) undergo an intensive rehabilitation program generally consisting of 3 hours of therapy per day at least 5 days a week with multiple therapy disciplines; 2) is medically stable and expected to benefit significantly, with measurable functional improvement from active participation within a prescribed period of time; 3) is supervised by a rehabilitation physician with face-to-face visits at least 3 days a week; and 4) undergoes the program with a coordinated, interdisciplinary team approach 1. These guidelines are mandatory for CMS-affiliated payors and other payors tend to follow them. The goal of acute inpatient rehabilitation is to provide concurrent medical and rehabilitative care for patients 2.

In a systematic review, the adherence rate for supervised physical therapy in outpatient settings for patients with cancer was greater than 64% 3. There is a gap in knowledge regarding adherence to acute inpatient rehabilitation in patients with cancer, especially related to medical complications, which have been noted to be high at our institution 4. Therefore, the purpose of this study was to assess adherence and describe the reasons for not participating fully during acute inpatient cancer rehabilitation to pioneer this topic in the medical literature.

## Methods

This is a secondary analysis of a retrospective study that included 165 patients, whose demographic and clinical characteristics along with data collection procedures were described and published previously (see Table S1) 4. Approval for data collection was obtained from the institutional review board.

Progress notes from rehabilitation clinicians (physical therapists, occupational therapists, speech & language pathologists, physical medicine & rehabilitation physicians, and their advanced practice providers) were reviewed to measure adherence and identify reasons for incomplete therapy sessions, which were defined as fewer than 45 minutes of participation out of 60 minutes scheduled per rehabilitation session. At our institution, the rehabilitation sessions are provided as 1-hour sessions 3 times a day on weekdays and an additional 1-hour session on a weekend day. Sessions on weekends, holidays, admission days, and discharge days were excluded from this study because patients did not consistently undergo 3 hours of rehabilitation sessions on those days. The duration of each rehabilitation session is documented in each progress note by physical and occupational therapists. Speech and language pathology sessions were assumed to be complete sessions (unless explicitly stated as a missed session) as their durations were not recorded.

We assessed adherence by measuring the frequency and proportion of patients who were noted to have 1 or more incomplete rehabilitation sessions on days when they should have had 3 hours of rehabilitation sessions. We did not assess adherence based on the total number of rehabilitation sessions since patients had an additional rehabilitation session on a weekday (6^th^) day beyond the requirement of 3 hours of rehabilitation sessions per day at least 5 days out of the week. In identifying reasons for incomplete rehabilitation sessions, problems identified as moderate or high risk of complications in the medical decision-making section of CMS guidelines 5 were defined as medical complications 4. The details regarding these complications were described previously 4. Data were managed using Research Electronic Data Capture for descriptive statistical analysis.

## Results

The total cohort of 165 patients had a median length of stay of 11 days (interquartile range of 8-14) in acute inpatient rehabilitation. Among these patients, there were 61 (37%) patients who had metastatic solid tumors and 69 (42%) who had solid tumors without metastasis. Baseline demographic and clinical characteristics including neoplasm types were previously published (see Table S1) 4. There were 91 (55%) surgical admission patients and 74 (45%) medical admission patients. These patients were transferred to acute inpatient rehabilitation due to neoplasm and/or its treatment-related deconditioning and neuromuscular deficits. Co-morbidities included the following: hypertension 80 (49%), diabetes mellitus 36 (22%), depression 32 (19%), obesity 21 (13%), chronic pulmonary disease 17 (10%), renal failure 15 (9%), congestive heart failure 7 (4%), liver disease 4 (2%), peripheral vascular disease 3 (2%), peptic ulcer disease 3 (2%), and rheumatologic disease 1 (0.5%).

Seventy-eight out of 165 (47%) patients had 1 or more incomplete rehabilitation sessions due to medical complications (Table 1), with a median of 2 (interquartile range 1-4) incomplete sessions. Most of the medical complication management involved symptom management 93 (39%), with interventions for pain management 47 (20%) being most frequent. We noted other reasons for incomplete rehabilitation sessions in 146 (89%) patients (Table 2) with a median of 3 (interquartile range of 2-4) incomplete rehabilitation sessions. Of these other reasons for incomplete rehabilitation sessions, the most frequent were due to patient-reported symptoms 158 (32%), with fatigue 115 (24%) being most frequent. The median total number of days with fewer than 3 hours of rehabilitation sessions was 2 (interquartile range 1-3) in the entire cohort.

## Discussion

To our knowledge, this is the first report of rates of adherence and reasons for non-adherence to acute inpatient rehabilitation among patients with cancer. We noted a high frequency (47%) of patients having incomplete rehabilitation sessions due to medical complications and a very high frequency (89%) of patients having incomplete rehabilitation sessions due to other reasons. This resulted in a median of 2 days with less than 3 hours of rehabilitation sessions. Incomplete rehabilitation sessions can prolong rehabilitation length of stay and decrease the effectiveness of acute inpatient rehabilitation.

In order to maximize patient adherence to rehabilitation sessions, several interventions can be considered. Some interventions such as optimization of medical comorbidities, pharmacological management for improved symptom control, and coordination of care with acute care oncology services to ensure medical stability, can be applied before transfer to (and during) acute inpatient rehabilitation. Ideally, these interventions should be performed before starting an intensive acute inpatient rehabilitation program to minimize interruptions during rehabilitation participation. Other steps, such as improvement of documentation of incomplete rehabilitation sessions, communication to allow for flexibility with schedules, and coordination of rehabilitation sessions and planned medical care (i.e. providers' assessments and care, procedures, etc.), can help improve adherence to rehabilitation sessions.

Although an acute inpatient rehabilitation program generally consists of at least 3 hours of rehabilitation per day at least 5 days a week, in well-documented cases, it can be provided as at least 15 hours of intensive rehabilitation therapy over 7 consecutive days 1. Also, rehabilitation can be given in six 30-minute sessions to total 3 hours of rehabilitation sessions per day. These alternative schedules can be considered, if feasible, at a facility with enough therapists.

When patients are transferred from acute medical care to acute inpatient rehabilitation, their medical condition should be stable 1 and they should be able to tolerate 3 hours of rehabilitation daily to prevent readmission to acute medical care services 6. Although some studies 4, 7-9 have evaluated risk factors for return to acute care services and our previous study have evaluated medically unstable conditions 4 during acute inpatient rehabilitation in patients with cancer, further studies are needed to delineate the various and extensive unstable medical conditions as well as other reasons that prevent full participation during acute inpatient rehabilitation. Besides treatment time, the content of rehabilitation activities and patients' tolerance to those activities are important factors to be considered when evaluating overall functional gain.

## Limitations

This study is limited by its small sample size and assessment at a single institution. Because this was a retrospective study, it depended on the accuracy of the rehabilitation clinicians' progress notes. Factors such as the phase of oncological treatment, patients' motivation or level of education, and living conditions were not documented consistently in the majority of the studied population before transfer to acute inpatient rehabilitation. Unlike physical and occupational therapy progress notes, speech and language pathology progress notes did not routinely document session duration. Therefore, we could have underestimated the true rate of adherence and overall assessment of various reasons for non-adherence. Nevertheless, this pilot study provides a framework for understanding adherence to acute inpatient rehabilitation.

## Conclusion

Among patients with cancer undergoing acute inpatient rehabilitation, the number of patients unable to adhere to 1-hour long rehabilitation sessions to complete 3 hours of rehabilitation sessions a day was high. Further research is needed to assess adherence and reasons for non-adherence and improve participation to maximize functional gains from acute inpatient rehabilitation.

## Supplementary Material

Supplementary table S1.

## Figures and Tables

**Table 1:** Medical complications^a^ resulting in incomplete rehabilitation sessions in 165 patients with cancer

Medical complication	Number of incomplete therapy session, No = 240
**Symptom management**	93 (39)
Pain	47 (20)
Diarrhea	17 (7)
Dyspnea	16 (7)
Nausea	10 (4)
Anxiety/Depression	3 (1)
**Neurological**	38 (16)
Focal deficits	13 (5)
Seizure	9 (4)
Acute altered mental status	9 (4)
Cognitive impairment	4 (2)
Complicated headache	3 (1)
**Cardiovascular**	35 (15)
Hypotension	13 (5)
Orthostasis	10 (4)
Tachycardia	7 (3)
Hypertension	3 (1)
Acute chest pain	2 (0.8)
**Infectious**	28 (12)
Fever work up	19 (8)
Upper respiratory infection	3 (1)
Surgical site infection	2 (0.8)
Urinary tract infection	2 (0.8)
Pneumonia	1 (0.4)
Mandibular infection	1 (0.4)
**Gastrointestinal**	19 (8)
Ileus	6 (3)
Pancreatitis	4 (2)
Nutritional disorder	4 (2)
Esophagogastroduodenoscopy	2 (0.8)
Rectal bleeding	2 (0.8)
Paracentesis	1 (0.4)
**Hematological**	9 (4)
Anemia	7 (3)
Thrombocytopenia	1 (0.4)
Hematoma	1 (0.4)
**Genitourinary**	5 (2)
Hemodialysis	2 (0.8)
Lower urinary tract symptom	2 (0.8)
Genitourinary tract fistula	1 (0.4)
**Musculoskeletal**	5 (2)
Joint instability	3 (1)
Lower extremity edema	2 (0.8)
**Eye complaints**	4 (2)
**Integumentary**	3 (1)
Surgical wound care	2 (0.8)
Rash	1 (0.4)
**Endocrine/hypoglycemia**	1 (0.4)

^a^Medical complications related to incomplete rehabilitation sessions occurred in 78 of 165 (47%) patients.

**Table 2:** Reasons^a^ for incomplete rehabilitation sessions other than medical complications in 165 patients with cancer

Other reasons^b^	Number of incomplete therapy sessions, No. = 499^a^
**Symptoms^c^**	158 (32)
Fatigue	118 (24)
Pain	25 (5)
Nausea/vomiting	8 (2)
Dyspnea	6 (1)
Dizziness	1 (0.2)
**Unspecified**	148 (30)
**Bladder/bowel care**	43 (9)
**Schedule conflict**	40 (8)
**Clinician assessment**	28 (6)
**Deferred by patient**	34 (7)
**Procedure**	26 (5)
**Follow-up imaging**	21 (4)
**Medication administration**	11 (2)
**Surgical wound care**	9 (2)
**Medical problems^c^**	6 (1)

^a^One hundred forty-six of 165 (88%) patients had incomplete rehabilitation sessions unrelated to the medical complications already noted in Table 1. ^b^Some incomplete therapy sessions were due to multiple reasons. ^c^These were symptoms or medical problems that did not result in any changes in interventions significant enough to be considered medical complications.

## Abbreviations

CMS: Centers for Medicare & Medicaid Services
