# Supplementary material for: Analysis of Adherence to Acute Inpatient Rehabilitation in Patients with Cancer
Source: J Cancer. 2021 Aug 20;12(20):5987–90. doi: 10.7150/jca.61010 (PMC8425205; doi:10.7150/jca.61010)
Supplement: Supplementary file 1 — Supplementary table S1. [file jcav12p5987s1.pdf]

Table S1: Demographic and clinical characteristics of patients who underwent acute inpatient rehabilitation (n=165)

	**Total**<br>**n = 165 (%)**
	**Median (IQR[a])**
Age	64 (55-71)
Hospital length of stay in days before transfer to rehabilitation	11 (8-18)
Acute inpatient rehabilitation length of stay in days	11 (8-14)
Hospital length of stay total number of days	26 (20-36)
Race	**n (%)**
· Caucasian/White	118 (72)
· African American/Black	20 (12)
· Asian	7 (4)
· Other	20 (12)
Hispanic ethnicity	26 (16)
Female gender	84 (51)
Married	117 (71)
Insurance	
· Medicare	94 (57)
· Commercial	58 (35)
· Self-pay	7 (4)
· Medicaid	4 (2)
· Other governmental	2 (1)
Neoplasm type	
· Brain and other nervous systems	39 (24)
· Hematologic and lymphatic	34 (21)
· Bones and connective tissues	25 (15)
· Genitourinary	19 (12)
· Breast	12 (7)

· Respiratory	12 (7)
· Digestive system	9 (6)
· Skin	6 (4)
· Others[b]	6 (4)

[a]Results as median (IQR 25-75)

[b]Included oral cavity and pharynx, endocrine, and other soft tissue neoplasms.
